# Identification of Inter-Organ Vascular Network: Vessels Bridging between Organs

**DOI:** 10.1371/journal.pone.0065720

**Published:** 2013-06-14

**Authors:** Madoka Omae, Norio Takada, Shohei Yamamoto, Hiroyuki Nakajima, Thomas N. Sato

**Affiliations:** 1 Graduate School of Biological Sciences, Nara Institute of Science and Technology, Nara, Japan; 2 Department of Cell Biology, National Cerebral and Cardiovascular Center Research Institute, Osaka, Japan; 3 Department of Biomedical Engineering, Cornell University, Ithaca, New York, United States of America; 4 Centenary Institute, Sydney, Australia; Peking University, China

## Abstract

Development and homeostasis of organs and whole body is critically dependent on the circulatory system. In particular, the circulatory system, the railways shuttling oxygen and nutrients among various organs, is indispensible for inter-organ humoral communication. Since the modern view of the anatomy and mechanics of the circulatory system was established in 17^th^ century, it has been assumed that humoral factors are carried to and from organs via vascular branches of the central arteries and veins running along the body axis. Over the past few decades, major advances have been made in understanding molecular and cellular mechanisms underlying the vascularization of organs. However, very little is known about how each organ is linked by vasculature (i.e., inter-organ vascular networks). In fact, the exact anatomy of inter-organ vascular networks has remained obscure. Herein, we report the identification of four distinct vessels, V1^LP^, V2^LP^, V3^LP^ and V4^LP^, that bridge between two organs, liver and pancreas in developing zebrafish. We found that these inter-organ vessels can be classified into two types: direct and indirect types. The direct type vessels are those that bridge between two organs via single distinct vessel, to which V1^LP^ and V2^LP^ vessels belong. The indirect type bridges between two organs via separate branches that emanate from a stem vessel, and V3^LP^ and V4^LP^ vessels belong to this type. Our finding of V1^LP^, V2^LP^, V3^LP^ and V4^LP^ vessels provides the proof of the existence of inter-organ vascular networks. These and other yet-to-be-discovered inter-organ vascular networks may facilitate the direct exchange of humoral factors that are necessary for the coordinated growth, differentiation and homeostasis of the connected organs. It is also possible that the inter-organ vessels serve as tracks for their connected organs to follow during their growth to establish their relative positions and size differences.

## Introduction

The intricate vascular network of the circulatory system is critical for growth and homeostasis of cells, organs and whole body [1]. The circulatory system delivers oxygen and nutrients to cells and organs, and drains their wastes. It is also the primary route to deliver hormones to various organs to regulate their growth and homeostasis. Furthermore, the circulatory system mediates local paracrine communication at the tissue level during development and regeneration by reciprocal chemical interaction between cells consisting of the circulatory system and tissues/organs.

The anatomical and functional recognition of the circulatory system dates back to 300s BC when Aristotle made the careful observation and documentation of the vascular anatomy, and the modern view of the circulatory system was established by William Harvey in early 17^th^ century [2]. The circulatory system consists of arteries, veins and microvasculature. Each organ is fed and drained by vascular branches emanating from large central arteries and veins running along the body axis [1]. Therefore, it is essential for each organ to attract their vascularization during development to establish their circulation [3]–[5]. Each organ is vascularized by vasculogenesis and/or angiogenesis and vascular remodeling [3]–[5]. These processes are in part mediated by paracrine factors exchanged among cells consisting of the vascular system and of the tissue microenvironment [6]–[13]. These reciprocal paracrine interactions between vessels and tissue microenvironment are indispensible for development and regeneration of many organs. Advances made in this area of investigation have significantly contributed to our extensive understanding of the mechanism underlying the formation of intra-organ vascular networks.

In contrast to our relatively clear understanding of the anatomy and function of intra-organ vascular networks, very little is known about inter-organ vascular networks. Since our modern view of the circulatory system was established in 17^th^ century, it has been assumed that each organ is vascularized by branches originating from central arteries and veins that run along the body axis, through which humoral factors are exchanged among various organs [1]. However, the vascular anatomy bridging between organs (i.e., inter-organ vascular networks) has remained poorly understood. In fact, the proof of the presence of vessels bridging between organs has been lacking.

To address this long-standing question, we used developing zebrafish to examine these putative inter-organ vascular connections using confocal and two-photon microscopy imaging techniques. Herein, we report the identification of the inter-organ vessels by direct visualization. We discovered four vessels, V1^LP^, V2^LP^, V3^LP^ and V4^LP^ that bridge between two organs, pancreas and liver, in developing zebrafish.

## Results and Discussion

The entire vascular system in developing zebrafish was visualized by using *Tg(fli1:egfp)^ y1^* transgenic zebrafish, a pan-vascular-endothelial fluorescent reporter line [14], [15]. The vascular connection was further confirmed by intracardiac injection of Qtracker 655 non-targeted quantum dots (QD655) that highlight nearly all perfused vasculature in the body. Liver and pancreas were specifically visualized by using *Tg(lfabf:DsRed;elaA:egfp)* transgenic zebrafish line [16]–[18]. In some experiments a newly generated fluorescent-reporter line for pancreas and liver, *Tg(elaA:TagRFP-Eco.NfsB), Tg(elaA:TagRFP-Eco.NfsB;fli1:egfp), and Tg(lfabf:TagRFP-Eco.NfsB;fli1:egfp)* were also used. Imaging analyses of *Tg(lfabf:DsRed;elaA:egfp)* show that pancreas and liver become visible by 2–3 days post fertilization (dpf) and 3–4 dpf, respectively (Fig. S1). Liver continues to grow in both anterior (i.e., away from pancreas) and posterior (i.e., towards pancreas) directions, and pancreas primarily in dorsal and ventral directions, but also in anterior and posterior directions to a lesser extent (Fig. S1).

**Figure 1:**
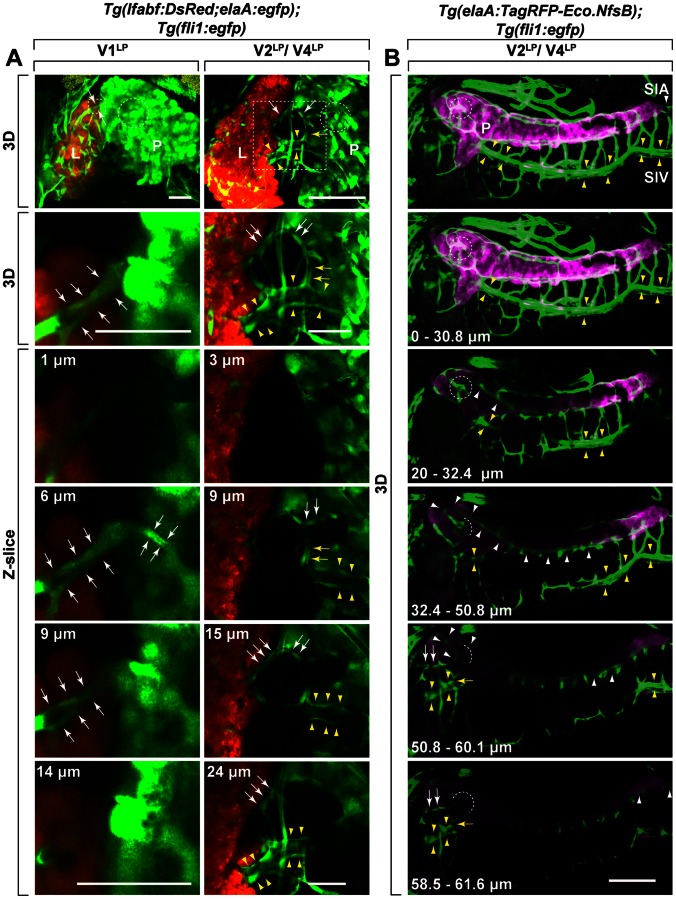
Identification of V1^LP^, V2^LP^ and V4^LP^, as inter-organ vessels bridging between liver and pancreas. Three distinct vessels (V1^LP^, V2^LP^, V4^LP^) were found to bridge between liver (L) and pancreas (P). **A.** The 3D rendered two-photon miscroscopy images (top two panels: 3D) and their serial optical sections (bottom panels: Z-slice) of *Tg(lfabf:DsRed;elaA:egfp);Tg(fli1:egfp)* at 5 dpf and 6 dpf. The second row 3D panels are the higher magnification of the indicated area (dotted rectangle) in the first row 3D panels. The bottom four panels are Z-slices of the areas shown in second row 3D panels. The depth of each slice is indicated as µm at the top left in each panel of Z-slice. V1^LP^ and V2^LP^ vessels are sandwiched between white arrows and indicated by white arrows, respectively. V4^LP^ vessel is indicated by yellow arrows and supraintestinal veins (SIV) is sandwiched between yellow arrowheads). The islet of Langerhans is indicated (dotted circle in each panel). The islet of Langerhans, consisting of endocrine cells, was identified as an area devoid of EGFP reporter signals driven by the exocrine pancreas-specific *elaA* promoter (see Fig. 2). In the first column, V1^LP^ vessel (sandwiched white arrows) bridging between liver (L) and pancreas (P) can be clearly seen. Examining the series of Z-slices confirms that V1^LP^ invades into liver tissue. In the second column, V2^LP^ (white arrows) and V4^LP^ (yellow arrows and sandwiched between yellow arrowheads) vessels bridging between liver (L) and pancreas (P) are shown. A connection between V4^LP^ and V2^LP^ appears to exist (yellow arrows in the 2^nd^ panel). Liver (L: orange); endocrine pancreas (P: green); *fli1^+^* vessels (green). Scale bars: 30 µm. **B.** V2^LP^ (white arrows) and V4^LP^ (yellow arrows and sandwiched between yellow arrowheads) vessels originate from supraintestinal arteries (SIA) and supraintestinal veins (SIV), respectively. The origins of V2^LP^ and V4^LP^ vessels were followed by using *Tg(elaA:TagRFP-Eco.NfsB);Tg(fli1:egfp)* at 6 dpf. The 3D-rendered Z-stack confocal microscopy images of several Z-slices (depth of ranges is indicated at the bottom left in each panel) are shown in series. *Fli1^+^* vessels and *elaA^+^* exocrine pancreas are shown as green and magenta, respectively. By following the SIA (white arrowheads) in each Z-stack, V2^LP^ is found to originate from vascular plexus at the islet of Langerhans (dotted circle) that is formed by branches of SIA. The 4^th^ and 5^th^ Z-stack panels show that vascular plexus (white arrowheads) at the islet of Langerhans (dotted circle) is formed by branches of SIA. In the 5^th^ Z-slice panel, the direct connection between this SIA-derived vascular plexus at the islet of Langerhans (dotted circle) and V2^LP^ (white arrows) can be seen. Following the vessels pointed by yellow arrows in each Z-stack demonstrate that V4^LP^ vessel is a part of SIV running ventral to pancreas. Supraintestinal artery (SIA) and supraintestinal vein (SIV) are indicated by white arrowheads and sandwiched between yellow arrowheads, respectively. Scale bars: 100 µm.

**Figure 2:**
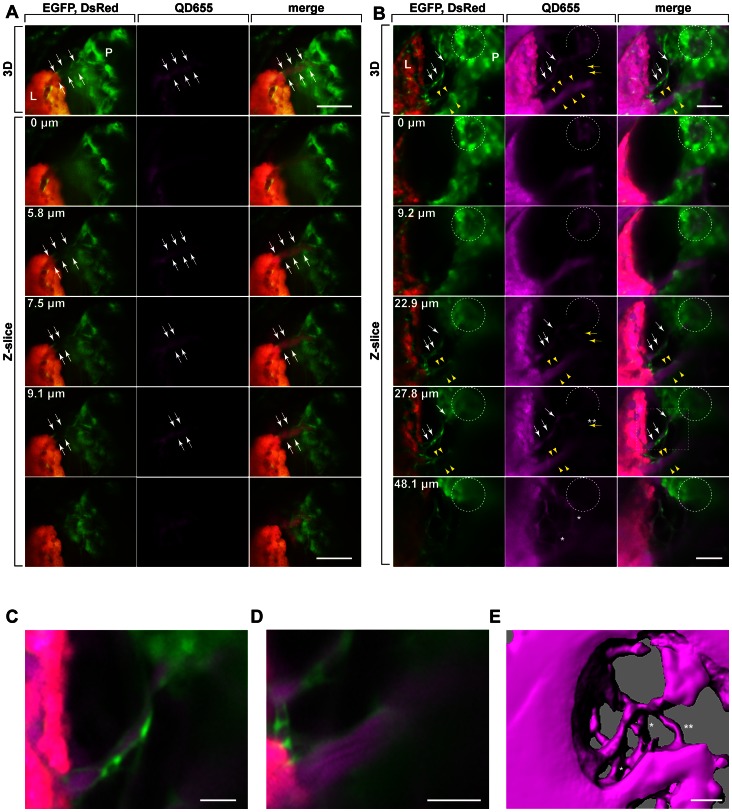
Delineation of V1^LP^, V2^LP^ and V4^LP^ vascular connections by quantum dots. QD655 injected *Tg(lfabf:DsRed;elaA:egfp);Tg(fli1:efgp)* larva is shown. The 3D rendered confocal microscopy images (the top panel: 3D) and their serial optical sections (bottom panels: Z-slice) of *Tg(lfabf:DsRed;elaA:egfp);Tg(fli1:egfp)* at 6 dpf are shown. The bottom five panels are Z-slices of the 3D rendered images shown at the top panel. The depth of each slice is indicated as µm at the top left in each panel of Z-slice. V1^LP^ and V2^LP^ vessels are sandwiched between white arrows and outlined by white arrows, respectively. V4^LP^ vessel is outlined by yellow arrows and sandwiched between yellow arrowheads. The islet of Langerhans is indicated (dotted circle in each panel). The islet of Langerhans, consisting of endocrine cells, was identified as an area devoid of EGFP reporter signals driven by the exocrine pancreas-specific *elaA* promoter. The left, middle and right panels in **A** and **B** show EGFP (green: *fli1^+^* vessels, *elaA^+^* exocrine-pancreas)/DsRed (orange: *lfabf^+^* liver), QD655 (magenta) and merged images, respectively. **A.** The QD655 perfused V1^LP^ vessel. The *fli1^+^* V1^LP^ vessel (green) is perfused with Q655 (magenta) albeit only faintly. **B.** The QD655 perfused V2^LP^ and V4^LP^ vessels. V2^LP^ and V4^LP^ vessels are outlined by white and yellow arrows, respectively. By following each serial Z-slices, the direct connection of V2^LP^ vessel (white arrows) to vascular plexus at the islet of Langerhans (dotted circle) is discernable. V4^LP^ vessel (yellow arrows and sandwiched between yellow arrowheads) is clearly connected to intrahepatic vasculature. There are three branches (two indicated by *, one indicated by**) stemming from V4^LP^ that appear to bridge between V2^LP^ and V4^LP^ vessels. By following the serial Z-slices, it is clear that two of these QD655 perfused vessels indicated by * become more discernable as V2^LP^ becomes fainter (compare the 4^th^ and 5^th^ Z-slices in the QD655 column), suggesting that those two indicated by * are not directly linked to either V2^LP^ or V4^LP^ vessels. Instead, they cross behind V2^LP^ vessel. In contrast, the one indicated by ** appears to be on the same Z-slice plane as V2^LP^ vessel (compare the 4^th^ and 5^th^ Z-slices in the QD655 column), suggesting that this vessel (**) is a branch that directly bridge between V2^LP^ and V4^LP^ vessels. **C.** Higher magnification of the V2^LP^ vessel showing co-localization of *fli1^+^* vascular endothelial cells (green) and QD655 signal (magenta) (of the 4^th^ panel of the merge column as indicated by dotted rectangle). **D.** Higher magnification of the V4^LP^ vessel showing co-localization of *fli1^+^* vascular endothelial cells (green) and QD655 signal (magenta) (of the 3^rd^ panel of the merge column as indicated by dotted rectangle). **E.** IsoSurface object image of QD655 perfused vessel connections of V2^LP^ and V4^LP^ and their branches. The QD655 perfused vessel image was treated by surface rendering method and IsoSurface object was built (Threshold = 12). In this surface rendered image, all three branches (? and **) stemming from V4^LP^ vessel appear to be fused to V2^LP^ vessel. However, as demonstrated by Z-slices shown in C., two (?) are crossing behind V2^LP^ vessel, and the one (**) fuses with V2^LP^ vessel. Scale bars: A, B: 50 µm; C–E: 25 µm.

A triple transgenic line, *Tg(lfabf:DsRed;elaA:egfp);Tg(fli1:egfp)* and a double transgenic line, *Tg(elaA:TagRFP-Eco.NfsB);Tg(fli1:egfp)* were used to highlight liver(DsRed)/pancreas(EGFP)/vessels(EGFP) and pancreas(TagRFP)/vessels(EGFP), respectively (Fig. 1A, B). Three *fli1^+^* vessels, V1^LP^, V2^LP^ and V4^LP^, bridging between liver and pancreas were identified (Fig. 1A). V1^LP^ bridging between liver and pancreas was detected by 5 dpf (Fig. 1A). However, we found this vessel in 5 out of 15 larvae examined, suggesting that V1^LP^ is a transiently existing vessel, or it is a collateral-like vessel that varies among individual zebrafish. By 6 dpf, two other distinct vessels, V2^LP^ and V4^LP^ bridging between liver and pancreas were identified (Fig. 1A). Both V2^LP^ and V4^LP^ were found in all larvae that we examined (13/13 and 9/9 for V2^LP^ and V4^LP^, respectively). In *Tg(lfabf:DsRed;elaA:egfp);Tg(fli1:egfp)* line, both pancreas (*elaA:egfp*) and vessels (*fli1:egfp*) are indistinguishable within the pancreatic tissue. Thus, we examined the connection of these inter-organ vessels inside the pancreatic tissue by using *Tg(elaA:TagRFP-Eco.NfsB);Tg(fli1:egfp)* line in which vessels and pancreas are distinguishable by their differential fluorescence (Fig. 1B). Examination of series of optical sections derived from this transgenic line demonstrated that V2^LP^ originates from vascular plexus at the islet of Langerhans that is formed by branches of supraintestinal arteries (SIA) (Fig. 1B). In contrast, it was found that V4^LP^ vessel is a branch of supraintestinal vein (SIV) (Fig. 1B). Further details of vascular connections and the perfusion of the vessels were examined by intracardial infusion of QD655 into *Tg(lfabf:DsRed;elaA:egfp);Tg(fli1:egfp)* triple transgenic line (Fig. 2). This analysis demonstrated that V1^LP^, V2^LP^ and V4^LP^ vessels are perfused by QD655 (Fig. 2A, B). Only faint and somewhat truncated signal of QD655 was found in V1^LP^ vessel, suggesting its relatively incomplete lumenization (Fig. 2A). In contrast, intense QD655 signals were detected in both V2^LP^ and V4^LP^ vessels, indicating that they are completely perfused (Fig. 2B–D). Furthermore, the direct connection of V2^LP^ to vascular plexus at the islet of Langerhans was confirmed (Fig. 2B). We also found the existence of three vessels crossing between V2^LP^ and V4^LP^ vessels (Fig. 2B). Following the series of optical scans, one of them (*) was found to be directly bridging V2^LP^ and V4^LP^ vessels (Fig. 2B, E, and Video S1), while the other two (**) are not connected to either V2^LP^ and V4^LP^ vessels but instead they appear to cross behind these vessels (Fig. 2B, E, and Video S1).

**Figure 3:**
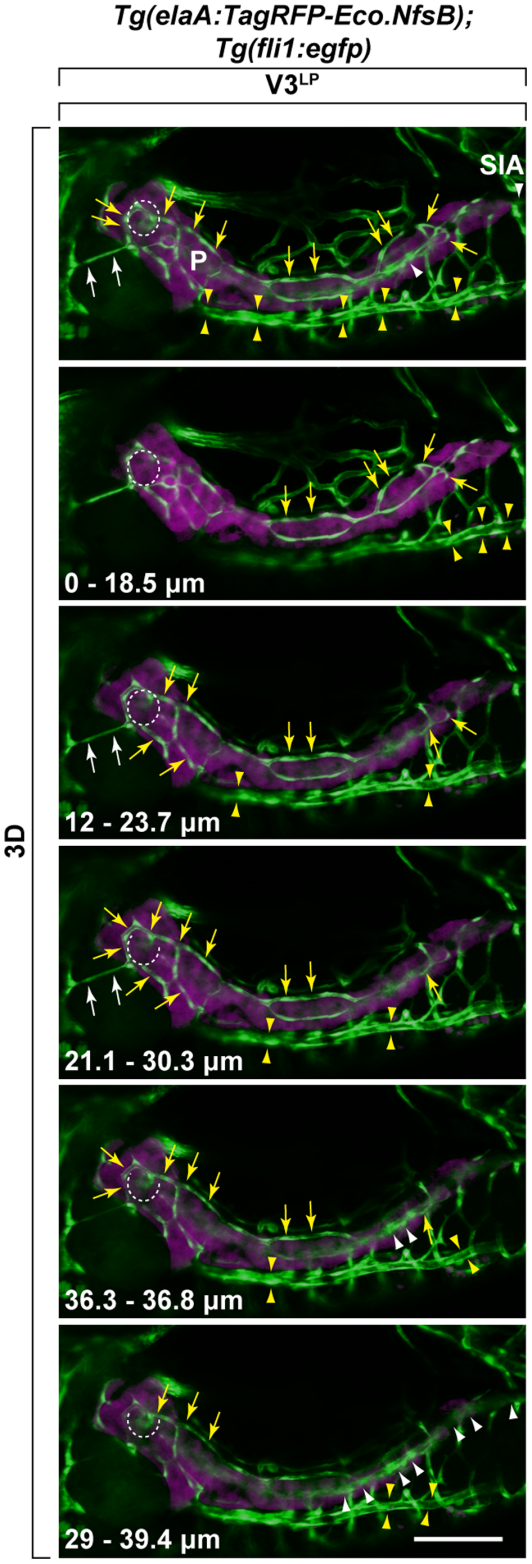
Identification of V3^LP^ vessel that bridges between liver and pancreas. V3^LP^ vessel originates from supraintestinal arteries (SIA). The origins of V3^LP^ was followed by using *Tg(elaA:TagRFP-Eco.NfsB);Tg(fli1:egfp)* at 5 dpf. The 3D-rendered Z-stack confocal miscroscopy images of several Z-slices (depth of ranges is indicated at the bottom left in each panel) are shown in series. *Fli1^+^* vessels and *elaA^+^* exocrine pancreas are shown as green and magenta, respectively. By following SIA (white arrowheads) in each Z-stack, V3^LP^ is found to be connected to a dorsal SIA branch (yellow arrows), to vascular plexus at the islet of Langerhans (dotted circle) that is linked to both dorsal (yellow arrows) and ventral (white arrowheads) SIA branches, and to SIV (sandwiched between yellow arrowheads), a part of which appears to be embedded inside pancreas. Supraintestinal artery (SIA): White arrowheads; Supraintestinal vein (SIV): Sandwiched between yellow arrowheads. Scale bars: 100 µm.

The other inter-organ vessel bridging between liver and pancreas, V3^LP^, was identified (Fig. 3). This vessel was found only rarely (3 out of 15 larvae examined), suggesting that it is a transiently existing or an individually variable collateral-like vessel. Following the series of optical sections, V3^LP^ appears to originate from one of the branches of SIA running over pancreas, a part of which runs inside the pancreatic parenchyma (Fig. 3). However, we cannot exclude a possibility that this SIA branch runs entirely inside the pancreatic parenchyma. The *fli1* promoter-driven EGFP expression of a part of this SIA branch appears to be over the TagRFP reporter signal driven by an *elaA* exocrine pancreas-specific promoter. Thus, it is possible that this EGFP signal is embedded within the basement membrane located over the exocrine cells close to the surface but still inside the pancreatic parenchyma [19]. V3^LP^ vessel appears to be associated with the liver parenchyma (Fig. 3), but its specific connection to intrahepatic vessels could not be revealed by the current method that we used.

**Figure 4:**
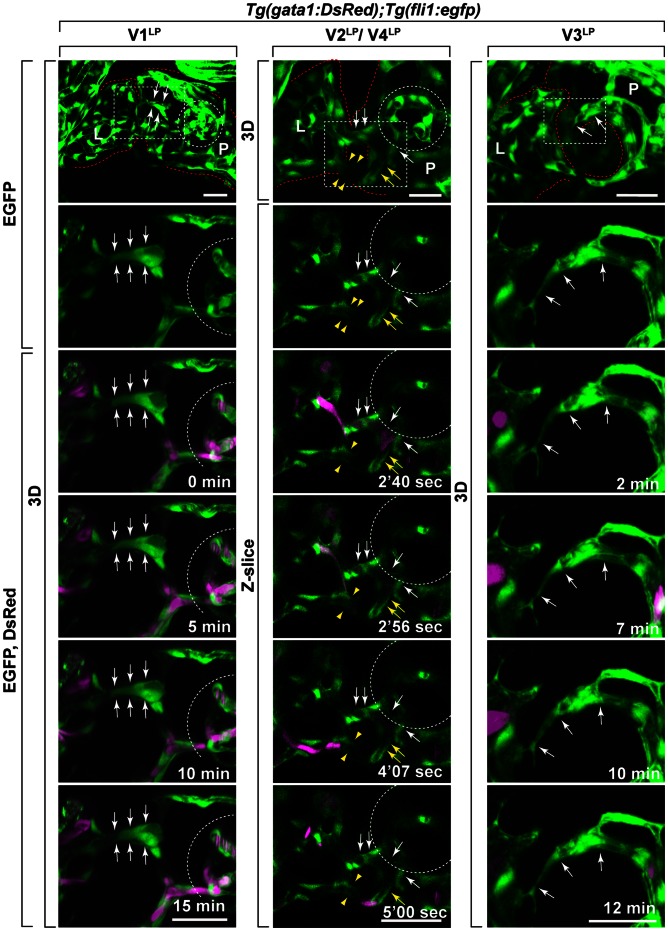
Identification of the circulation of *gata1^+^* blood cells through V2^LP^ and V4^LP^ inter-organ vessels. Time-lapse two-photon microscopy images of *Tg(gata1:DsRed);Tg(fli1:egfp)* larvae at 5 dpf (V1^LP^, V2^LP^, V4^LP^) and at 6 dpf (V3^LP^). No circulation of *gata1^+^* blood cells was observed within V1^LP^ or V3^LP^ vessels during the 15 min. or 14 min. imaging periods, respectively. In contrast, the circulation of *gata1^+^* blood cells was clearly observed both within V2^LP^ and V4^LP^ vessels during the 7 min. imaging period. The 3D rendered (3D) and/or Z-slices are shown. EGFP (green): *fli1^+^* vessels; DsRed (magenta): *gata1^+^* blood cells. The time-stamp (min: minute, sec: second) is indicated at the bottom right in each bottom panel. Scale bars: 30 µm.

The circulation of these inter-organ vessels was examined by using *Tg(gata1:DsRed);Tg(fli1:egfp)* [20] as *gata1* promoter drives the DsRed expression in blood cells (Fig. 4). This analysis revealed that the circulation of *gata1^+^* blood cells through both V2^LP^ and V4^LP^ vessels, but not through V1^LP^ or V3^LP^ (Fig. 4 and Video S2). These findings together with the QD655 perfusion results (Fig. 2) indicate that V2^LP^ and V4^LP^ vessels are blood-circulating vessels. In contrast, V1^LP^ and V3^LP^ are partially or completely lumenized, respectively, as shown with QD655 (Fig. 2), but not being circulated with blood cells as no *gata1^+^* blood cells were observed in these vessels (Fig. 4). Thus, V1^LP^ and V3^LP^ vessels are less frequently observed and are at least partially lumenized without circulating blood cells, suggesting that they are transiently forming inter-organ vessels.

**Figure 5:**
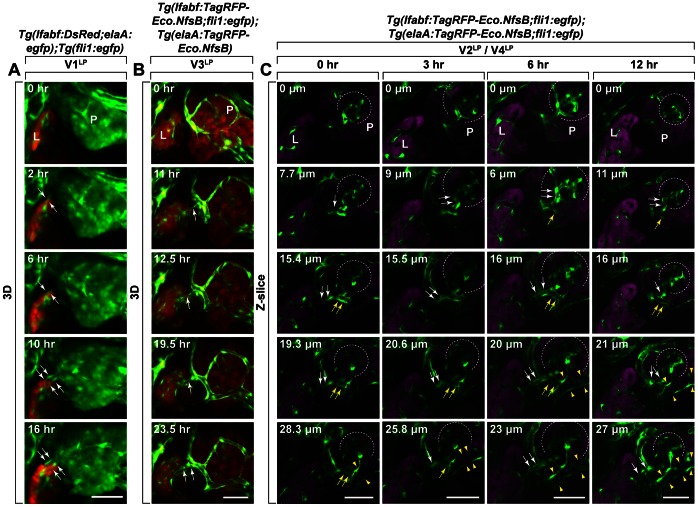
Dynamics of the formation of V1^LP^ and V3^LP^ vessels. The dynamic behavior of growing V1^LP^, V3^LP^, V2^LP^/V4^LP^ inter-organ vessels were analyzed by time-lapse confocal microscopy using *Tg(lfabf:DsRed;elaA:egfp);Tg(fli1:egfp)* (A) and *Tg(lfabf:TagRFP-Eco.nfsB;fli1:egfp);Tg(elaA:TagRFP-Eco.nfsB)* (B) larvae, respectively. A. Panels show images from right side of larva with dorsal up, and the images were captured beginning at about 4–4.5 dpf. The V1^LP^ vessel (EGFP: green) on the dorsal side (sandwhiched between white arrows) grows out of liver (DsRed: orange) and invades into pancreas (EGFP: green). B. Panels show images from right side of larva with dorsal up, and the images were captured beginning at about 4.5 dpf. The V3^LP^ vessel (EGFP: green) on the dorsal side (white arrows) grows out of pancreas (TagRFP: orange) and invades into liver (TagRFP: orange). Time (h) elapsed since the beginning of the sequence is indicated at the top right in each panel. C. Panels show images from right side of larva with dorsal up, and the images were captured beginning at about 4 dpf. The embryo was observed at 0 hr, 3^rd^ hr, 6^th^ hr and 12^th^ hr. V2^LP^ vessel (white arrows) branches off from the vascular plexus in Islet of Langerhans of pancreas was observed at 0 hr, but it connects with hepatic vascular network by 12^th^ hr. V4^LP^ vessel (yellow arrows and sandwiched between yellow arrowheads) that is linking between V2^LP^ (white arrows) and SIV(sandwiched between yellow arrowhead) was also observed. L: Liver, P: Pancreas. Scale bars: A, B: 40 µm; C: 30 µm.

These findings demonstrate the existence of two blood-circulating and two lumenized without blood-circulating inter-organ vessels between liver and pancreas. Next, we investigated the dynamics of the formation of these inter-organ vessels by time-lapse confocal microscopy imaging of *Tg(elaA:TagRFP-Eco.NfsB) and Tg(lfabf:TagRFP-Eco.NfsB;fli1:egfp)* triple transgenic zebrafish larvae (Fig. 5, Videos S3, S4). We found that V1^LP^ vessel emerges from liver at 4–4.5 dpf and invades into pancreas by 5.5 dpf (Fig. 5A, Video S3). In contrast, V3^LP^ vessel begins to sprout out of an SIA branch at pancreas by 4.5 dpf and completes the invasion into hepatic parenchyma by 5.5 dpf (Fig. 5B, Video S4). V2^LP^ vessel was found to growth out of vascular plexus of Islet of Langerhans of pancreas at around 4 dpf and link to hepatic vascular network by 4.5–5 dpf (Fig. 5C). V4^LP^ vessel was observed to link between V2^LP^ and SIV as a branch of V2^LP^ vessel between 4 dpf and 5 dpf (Fig. 5C). These findings highlight an example of two organs reciprocally sending off distinct sets of inter-organ vessels to link each other by the vascular network.

**Figure 6:**
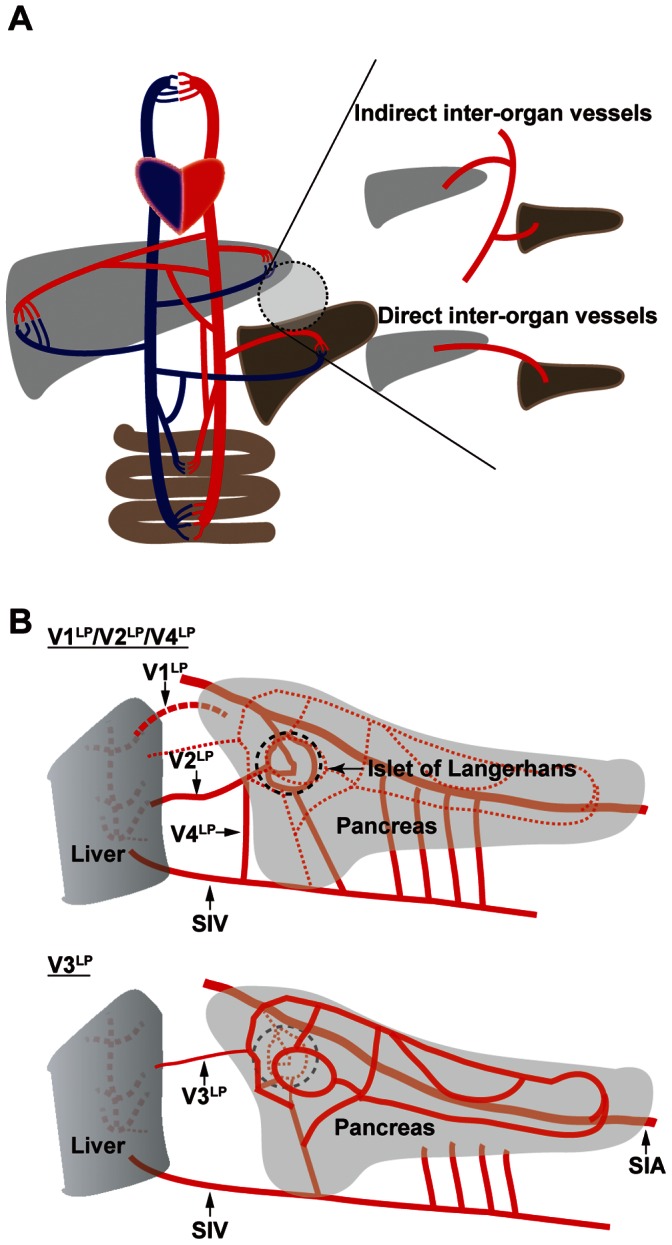
Schematic diagram of inter-organ vascular networks. **A.** Inter-organ vascular networks. Indirect inter-organ vessels bridge between two organs via two distinct branches of a “stem” vessel (top). In contrast, a direct inter-organ vessel bridges between two organs via a directly connecting distinct vessel (bottom). **B.** Identification of four vessels, V1^LP^, V2^LP^, V3^LP^ and V4^LP^, that bridge between liver and pancreas. V1^LP^ and V2^LP^ belong to the direct type as they bridge between liver and pancreas in a direct manner. In contrast, V3^LP^ and V4^LP^ belong to the indirect type as they bridge between these two organs in an indirect manner. In the V1^LP^/V2^LP^/V4^LP^ diagram (top), the intra-pancreatic vascular network indicated in the V3^LP^ diagram (bottom) is shown as dotted lines.

It has been assumed that humoral communication among organs in vertebrates is conferred by vascular branches of central arteries and veins, but the precise anatomical description of the vascular connections among organs was lacking (Fig. 6A). Herein we report the identification of inter-organ vascular networks, which bridge between two organs (Fig. 6A, B). In developing zebrafish, we demonstrated that there are four distinct vessels, V1^LP^, V2^LP^, V3^LP^ and V4^LP^, that bridge between liver and pancreas (Fig. 6B). V1^LP^ and V2^LP^ directly bridges two organs, thus belong to the direct type of inter-organ vessels (Fig. 6A, B). V3^LP^ originates from one of the SIA branches associated with the pancreatic parenchyma, and V4^LP^ is a branch of SIV that invades into liver with the connection to the pancreatic parenchyma via another branch stemming from SIV (Fig. 6B). Therefore, both V3^LP^ and V4^LP^ belong to the indirect type of inter-organ vessels (Fig. 6A, B). In conclusion, our result clearly demonstrates the presence of vessels that bridge between organs, and thus provides the definitive proof of the presence of inter-organ vascular networks.

What is the function of these and other yet-to-be-discovered inter-organ vascular networks? It is possible that the inter-organ humoral communication mediated by inter-organ vascular networks bestows coordinated development, differentiation and homeostasis on locally bridged organs. Organs may also exploit such inter-organ vessels as tracks for the direction and extent of their growth, processes that are critical for establishing their relative positions and sizes. Addressing these and other questions on this newly discovered type of vascular networks, i.e., inter-organ vascular networks, is anticipated to open up a myriad of new possibilities for the mechanism underlying inter-organ communication.

## Materials and Methods

### Zebrafish Breeding and Maintenance

Zebrafish fertilized eggs were collected in Egg raising buffer (0.06% artificial marine salt supplemented with 0.0002% methylene blue) and were raised at 28°C. To prevent pigmentation, embryos were raised in 1/3 Ringer's medium (1.67 mM HEPES, 38.7 mM NaCl, 0.97 mM KCl, 0.60 mM CaCl_2_, pH 7.2) containing 0.001% phenylthiourea (PTU) (Sigma). Staging (days post fertilization: dpf) of embryos and larvae were according to Kimmel *et al*. [21]. The published transgenic lines *Tg(lfabf:DsRed;elaA:egfp)*, *Tg(fli1:egfp)^ y1^,* and *Tg(gata1:DsRed)* were as previously described [15], [17], [20]. All animal protocols were approved by the Animal Care and Use Committee of Nara Institute of Science and Technology (Permit Number: 1234).

### Establishment of Tg(elaA:TagRFP-Eco.nfsB) and Tg(lfabf:TagRFP-Eco.nfsB) Zebrafish Line

The pancreatic exocrine-cell-specific elaA promoter sequence was kindly provided by Dr. Gong [18]. This promoter fragment was cloned in pT2AL200R150G (provided by Dr. Kawakami) [22]. The pTol2:elaA:egfp was constructed by replacing the EF1a-p-intron sequence by the elaA and L-FABP promoter sequence. The pTol2:elaA:TagRFP-Eco.NfsB was constructed by replacing the egfp sequence by TagRFP-Eco.NfsB sequence, which was amplified by PCR from EF1a:TagRFP:NTR construct (provided by Dr. Stainier) [23]. To produce both *Tg(elaA:TagRFP-Eco.NfsB)* and *Tg(lfabf:TagRFP-Eco.nfsB)* fish, pTol2:elaA:TagRFP-Eco.NfsB was injected into one-cell stage embryos with transposase mRNA. *Tg(lfabf:TagRFP-Eco.nfsB;fli1:egfp)* and *Tg(elaA:TagRFP-Eco.nfsB;fli1:egfp)* was generated by crossing *Tg(lfabf:TagRFP-Eco.nfsB)* and *Tg(elaA:TagRFP-Eco.nfsB)* with *Tg(fli1:egfp)^y1^*, respectively.

### Microscopy

At 4–6 dpf zebrafish larvae *Tg(lfabf:DsRed;elaA:egfp);Tg(fli1:egfp)* were anesthetized in 0.012% Tricaine and mounted in 1.0–1.5% low melting temperature agarose gel on glass-bottomed 35 mm Petri dishes. Just prior to taking the still-movie, the concentration of Tricaine was increased to 0.1%. Imaging by confocal microscopy was performed with 20 X dry (NA = 0.8) objective lens mounted on Zeiss LSM710 (Zeiss, Germany) and Z-stack images were recorded with an optical slice thickness. FV1000MPE multiphoton laser scannning microscope (Olympus) was used to obtain two-photon microscopy images with 920 nm excitation wavelength with 25 X water immersion objective lens (NA = 1.05). At 5–6 dpf zebrafish larvae *Tg(gata1:DsRed);Tg(fli1:egfp)* were anesthetized in 0.012% Tricaine and mounted in 1.0% low melting temperature agarose gel on 35 mm Petri dishes. FV1000MPE multiphoton laser scannning microscope (Olympus) was used to obtain two-photon microscopy images with 1000 nm excitation wavelength with 25 X water immersion objective lens (NA = 1.05). All Images were 3D rendered and analyzed using Imaris.

### Quantum Dots Injection

The Qtracker 655 non-targeted quantum dots (QD655) (Invitrogen) was used to visualize vascularture of zebrafish larvae at 4–6 dpf. For QD655 injection, an anesthetized larva was mounted in 1.0% low melting agarose, followed by the injection of 2 µM QD655 into the heart. After being allowed to perfuse for 0.5–6 hours, larvae were observed with Zeiss LSM710.

### Time-lapse Confocal Microscopy

Larvae used for imaging were anesthetized using Tricaine in 1.0–1.5% low melting temperature agarose and mounted on their sides in glass-bottomed 35 mm Petri dishes. Time-lapse images were captured using 20× dry (NA = 0.8) objective lens on Zeiss LSM710 microscope equipped with incubator to maintain larvae at 28.0°C. Z image stacks were collected every 5–30 min, and three-dimensional data sets were compiled using Imaris. For *Tg(gata1:DsRed)*; *Tg(fli1:egfp)* time-laps microscopy, larvae were anesthetized using Tricaine in 1.0% low melting temperature agarose and mounted on their sides in 35 mm Petri dishes. Time-lapse images were captured using 25× water immersion objective lens with 1000 nm excitation wavelength on FV1000MPE multiphoton laser scannning microscope (Olympus). Z image stacks or single slice were collected sequentially, and data sets were compiled using Imaris.

## Supporting Information

Figure S1 **Growth of liver and pancreas between 3 dpf and 6 dpf in developing zebrafish. A.** A zebrafish larva at 6 dpf imaged from the right side of the body showing the relative positions of liver (L), pancreas (P), gallbladder (Gb), swimbladder (Sb) and intestine (Int). The anterior and posterior sides on the left and right, respectively. **B.** The growth of pancreas and liver. The liver (orange) and pancreas (green) were visualized at 3, 4, 5 and 6 dpf using *Tg(lfabf:DsRed;elaA:egfp)*. Gb: Gallbladder. Scale bars: 100 µm.
(TIF)

Video S1 **IsoSurface object image of V2^LP^ and V4^LP^ vessels, and their branches.** IsoSurface object of the QD655 injected V2^LP^ and V4^LP^ vessels was generated using surface rendering of the volume data of the vessels and it is shown as a 360°-rotatable object. Surface rendering was performed using Imaris with threshold of 12.
(AVI)

Video S2 **The circulation of** ***gata1^+^*** **blood cells through V2^LP^ and V4^LP^ inter-organ vessels.** Time-lapse video of *Tg(gata1:DsRed;fli1:egfp)* larva at 5 dpf. The circulation of *gata1^+^* blood cells through V2^LP^ and V4^LP^ was captured by taking single-slice images at 5 frames per second.
(AVI)

Video S3 **Dynamics of V1^LP^ vessel formation.** Time-lapse video of *Tg(lfabf:DsRed;elaA:egfp);Tg(fli1:egfp)* larva beginning at about 4 dpf. The images are from the right side, focused on a portion between pancreas and liver. Dorsal is up and anterior left. The V1^LP^ vessels emerge from dorsal side of liver and invade into pancreas. Images were collected every 5 min for 30 hours, and the movie runs at 20 frames per second.
(AVI)

Video S4 **Dynamics of V3^LP^ vessel formation.** Time-lapse video of *Tg(lfabf:TagRFP-Eco.nfsB;fli1:egfp);Tg(elaA:TagRFP-Eco.nfsB)* larva beginning at about 4.5 dpf. The images are from the right side, focused on a portion between pancreas and liver. Dorsal is up and anterior left. The V3^LP^ vessels emerge from pancreas and invade into liver. Images were collected every 30 min for 24 hours, and the movie runs at 5 frames per second.
(AVI)
