# Supplementary figures and images for: Identification of Inter-Organ Vascular Network: Vessels Bridging between Organs
Source: PLoS One. 2013 Jun 14;8(6):e65720. doi: 10.1371/journal.pone.0065720 (PMC3683054; doi:10.1371/journal.pone.0065720)

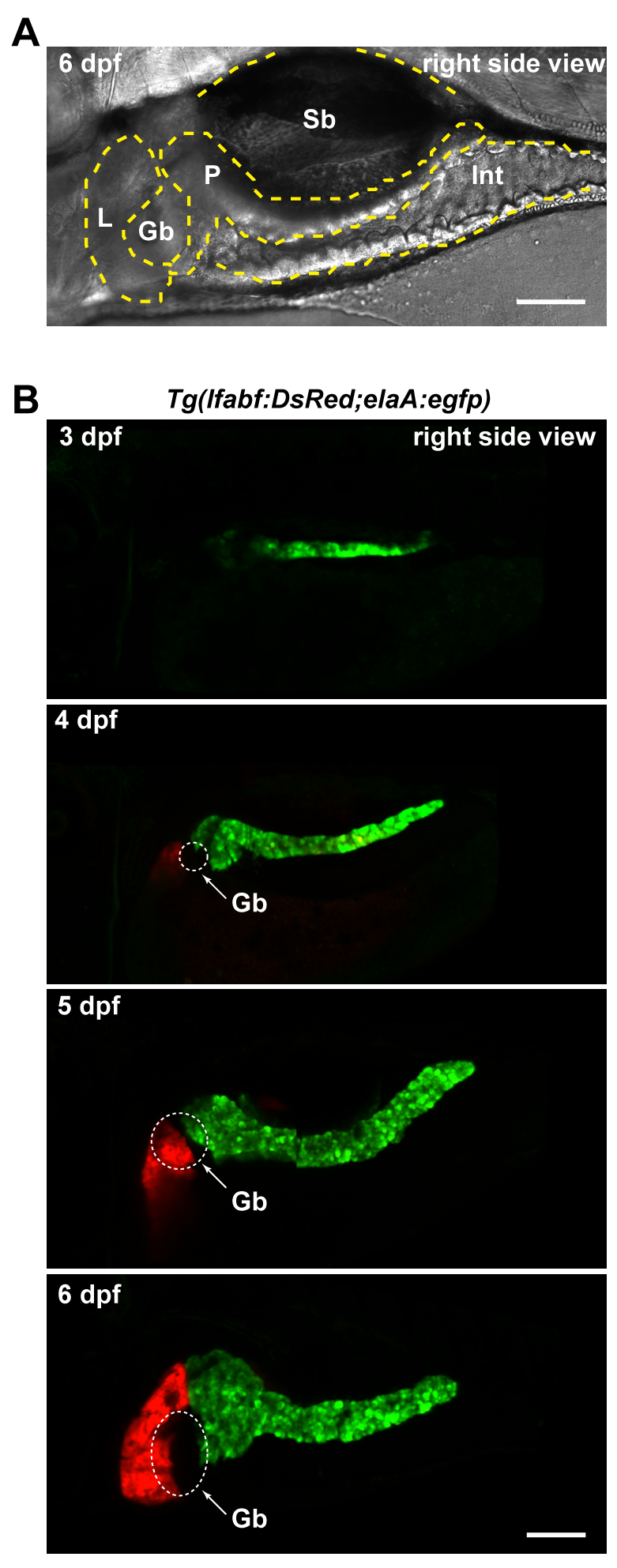

Supplement: Figure S1 — Growth of liver and pancreas between 3 dpf and 6 dpf in developing zebrafish. A. A zebrafish larva at 6 dpf imaged from the right side of the body showing the relative positions of liver (L), pancreas (P), gallbladder (Gb), swimbladder (Sb) and intestine (Int). The anterior and posterior sides on the left and right, respectively. B. The growth of pancreas and liver. The liver (orange) and pancreas (green) were visualized at 3, 4, 5 and 6 dpf using Tg(lfabf:DsRed;elaA:egfp). Gb: Gallbladder. Scale bars: 100 µm. (TIF) [file pone.0065720.s001.tif]
